# Association between atherogenic index of plasma in early pregnancy and risk of preeclampsia: a multicenter cohort study

**DOI:** 10.3389/fnut.2026.1849933

**Published:** 2026-06-18

**Authors:** Qiong Li, Yuping Duan, Chenyang Zhao, Ying Gu, Yanfei Qian, Chaoyan Yue

**Affiliations:** 1 Department of Obstetrics and Gynecology, The First People's Hospital of Chenzhou, Hengyang Medical School, University of South China, Chenzhou, China; 2 Department of Clinical Laboratory, Wusong Central Hospital, Baoshan, Shanghai, China; 3 Department of Obstetrics, Women's Hospital of Jiangnan University, Wuxi Maternity and Child Health Care Hospital, Wuxi, China; 4 Department of Clinical Laboratory, Children's Hospital of Fudan University, Shanghai, China; 5 Obstetrics & Gynecology Hospital of Fudan University, Shanghai Key Lab of Reproduction and Development, Shanghai Key Lab of Female Reproductive Endocrine Related Diseases, Shanghai, China

**Keywords:** atherogenic index of plasma, lipids, multicenter cohort study, preeclampsia, risk marker

## Abstract

**Background:**

This study aimed to investigate the relationship between atherogenic index of plasma (AIP) and preeclampsia (PE).

**Methods:**

This retrospective multicenter observational cohort study included 2,162 preeclampsia cases and 36,660 normal controls who met the eligibility criteria. AIP was measured before 20 weeks of gestation and calculated as log10 [TG (mmol/L)/HDL-C (mmol/L)]. Participants were divided into four groups according to AIP quartiles (Q1–Q4). Multivariable logistic regression, subgroup analysis, and smooth curve fitting were applied to examine the association between AIP and PE, as well as related adverse pregnancy outcomes.

**Results:**

After adjusting for confounding factors, multivariable logistic regression analysis revealed that participants in the Q3 and Q4 groups had significantly higher risks of PE and gestational diabetes mellitus with preeclampsia (GDM&PE) compared with those in the Q1 group. The magnitude of the independent association was modest: every 0.1 increase in AIP was associated with an 8% higher risk of PE (OR = 1.08, 95% CI: 1.05–1.10, *P* < 0.0001) and a 13% higher risk of GDM&PE (OR = 1.13, 95% CI: 1.07–1.19, *P* < 0.0001). Subgroup analyses further showed that the association between AIP and PE was not affected by maternal age, BMI, or parity. Additionally, a nonlinear dose-response association was observed between AIP and PE risk.

**Conclusion:**

In this retrospective observational cohort, elevated AIP in early pregnancy was associated with higher risks of PE and GDM&PE, although the adjusted effect sizes were modest. AIP may be considered a potential risk marker for PE and GDM&PE.

## Introduction

Preeclampsia (PE) is a common pregnancy complication characterized primarily by elevated blood pressure and proteinuria after 20 weeks of gestation, with an incidence rate of 5% to 8% (1, 2). PE poses significant risks to both maternal and fetal health, including complications such as placental abruption, multi-organ dysfunction, preterm birth, and fetal growth restriction (3, 4). Additionally, PE is considered a significant risk factor for future cardiovascular diseases in both mothers and offspring (5). In recent years, the incidence of PE has been increasing due to rising rates of maternal obesity and advanced maternal age (6). Given that its pathogenesis remains incompletely understood, effective prevention and treatment strategies are still lacking. Once typical clinical symptoms appear, the only effective intervention is termination of pregnancy, often resulting in preterm birth and low birth weight (LBW). Although PE exhibits clinical symptoms typically after 20 weeks of gestation, its underlying pathological changes occur before 20 weeks. Therefore, early identification of high-risk factors is crucial to prevent the development of PE and reduce adverse maternal and fetal outcomes.

Dyslipidemia increases the risk of atherosclerotic cardiovascular disease by inducing endothelial dysfunction (7). Similarly, the development of PE is also closely associated with dyslipidemia and impaired endothelial function. Previous studies have shown that lipid parameters such as total cholesterol (TC), triglycerides (TG), and high-density lipoprotein cholesterol (HDL-C) are associated with PE (8–10). However, the predictive value of individual lipid markers is limited. The plasma atherogenic index (AIP), a novel indicator of lipid metabolism, reflects the balance between pro-atherogenic and anti-atherogenic lipoproteins (11). By accounting for interactions among different lipid components, AIP more accurately reflects atherogenic lipid abnormalities compared to individual indicators (11), and has been established as an important parameter for evaluating lipid metabolic disorders (12, 13) and predicting cardiovascular risk (14). However, research on the relationship between AIP and PE remains limited. Some small-sample studies have reported significantly elevated AIP levels in patients with PE (15–17), but these studies were constrained by small sample sizes and insufficient statistical power. Moreover, in most of these studies, AIP was measured after the diagnosis of PE, limiting their ability to capture early pregnancy changes or determine whether AIP elevation precedes PE or occurs as a consequence. Additionally, potential confounding factors such as age, body mass index (BMI), and parity were not adequately controlled, making it difficult to assess the independent effect of AIP on the occurrence of PE.

To address the limitations of prior studies, this multicenter retrospective cohort study measured AIP before 20 weeks of gestation. Multivariate logistic regression analysis and subgroup analysis were conducted to adjust for potential confounders, while smooth curve fitting was used to explore the dose-response relationship between AIP and PE. We aimed to comprehensively investigate the association between early-pregnancy AIP and PE, as well as related adverse pregnancy outcomes, such as gestational diabetes mellitus with preeclampsia (GDM&PE), preterm birth, and LBW.

## Methods

### Study design

**Figure 1:**
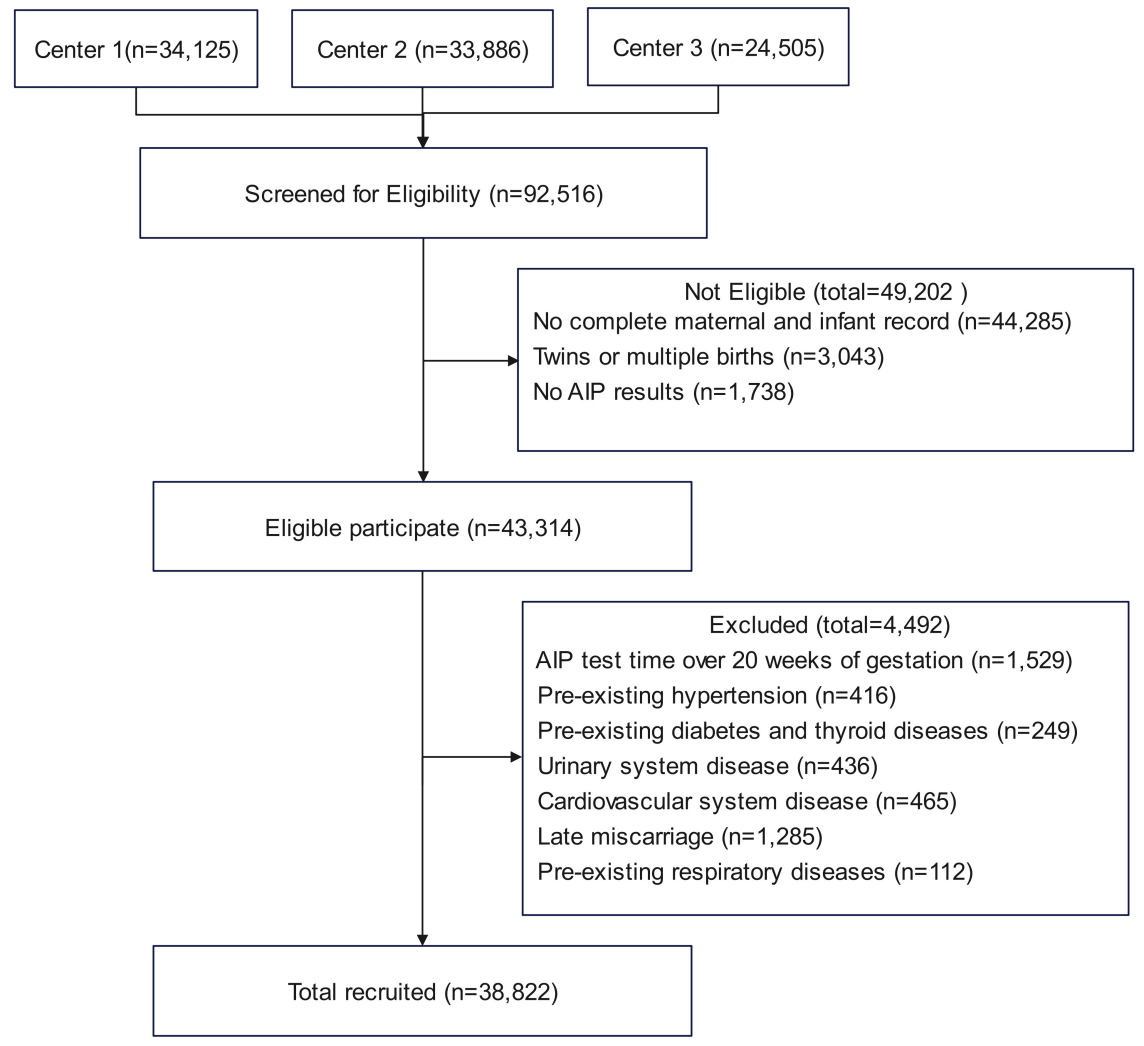
Flowchart of study participants.

This study was a multicenter retrospective observational cohort study. We consecutively screened singleton pregnant women who delivered at one of the three participating hospitals between January 2018 and June 2024: the Obstetrics and Gynecology Hospital of Fudan University, Huangpu Branch (Center 1), the Obstetrics and Gynecology Hospital of Fudan University, Yangpu Branch (Center 2), and the First People's Hospital of Chenzhou (Center 3). All eligible women with available first-prenatal-visit lipid measurements before 20 weeks of gestation and complete maternal and neonatal outcome records were included. The study finally included 38,822 singleton pregnant women, comprising 2,162 PE cases and 36,660 normal controls. Clinical and laboratory data were extracted from the Hospital Information System (HIS) and Laboratory Information System (LIS) by trained investigators using a unified data-extraction template. Across centers, TG and HDL-C were measured in fasting venous blood using routine enzymatic assays under each hospital laboratory's internal quality-control procedures; units were harmonized before AIP calculation. The same predefined diagnostic criteria and outcome definitions were applied across centers. The inclusion criteria were as follows: (1) age ≥20 years; (2) singleton pregnancy; (3) delivery at one of the participating hospitals. The exclusion criteria included initial detection of TG and HDL-C after 20 weeks of gestation, twin or multiple pregnancies, pre-pregnancy diabetes and thyroid dysfunction, chronic hypertension, cardiovascular diseases, urinary system diseases, respiratory system diseases, late miscarriage, and cases without complete maternal and neonatal records. Figure 1 illustrates the participant inclusion process. The study was approved by the Ethics Committees of the Obstetrics and Gynecology Hospital of Fudan University and Chenzhou First People's Hospital. The requirement for informed consent was waived for all participants, and the study adhered to the principles of the Declaration of Helsinki (as revised in Fortaleza, Brazil, October 2013).

### Variables and measurements

AIP was considered the exposure factor in this study. General clinical and demographic characteristics, including age, height, pre-pregnancy BMI, systolic blood pressure (SBP), diastolic blood pressure (DBP), smoking, alcohol consumption, family history of hypertension, family history of diabetes, and aspirin use were collected by experienced healthcare professionals. Aspirin use was recorded from medication information available at the first prenatal visit or in the electronic medical record; however, detailed indications and exact initiation timing were not uniformly available across centers. Height and weight were measured in a fasting state in the morning, with precision to 0.1 cm and 0.1 kg, respectively. Blood pressure was measured twice using an electronic sphygmomanometer after a 15-min rest, with the average of two readings recorded.

Laboratory data were collected during the first prenatal visit before 20 weeks of gestation. TG and HDL-C were reported in mmol/L, and AIP was calculated using the formula AIP = log10 [TG (mmol/L)/HDL-C (mmol/L)] (11). Because TG and HDL-C were expressed in the same units, the ratio used for AIP calculation was dimensionless. AIP values ranged from−0.74 to 0.58. Participants were stratified into quartiles based on AIP: Q1 (-0.74,−0.24), Q2 (-0.24,−0.08), Q3 (-0.08, 0.07), and Q4 (0.07, 0.58). Following delivery, maternal and neonatal clinical data, including gestational age at delivery and birth weight, were recorded.

Based on prior literature regarding risk factors for PE and clinical expertise, potential confounders were considered, including age, pre-pregnancy BMI, SBP, DBP, gestational week of testing, alanine aminotransferase (ALT), creatinine, fasting plasma glucose (FPG), aspirin use, family history of hypertension, family history of diabetes, smoking, alcohol consumption, *in vitro* fertilization (IVF), adverse pregnancy history, parity, and education level. Pre-pregnancy BMI was calculated as weight (kg) divided by height squared (m^2^) and categorized as underweight/normal (< 24 kg/m^2^) or overweight (≥24 kg/m^2^). Age was grouped as < 35 years and ≥35 years, consistent with clinical relevance and the ACOG/SMFM Obstetric Care Consensus on pregnancy at age 35 years or older (18). Adverse pregnancy history was defined as spontaneous abortion or significant obstetric complications. Education level was categorized into postgraduate, bachelor's degree or above, college diploma, high school, and less than junior high school.

### Outcomes and measurements

The primary outcome PE was defined as follows: normal blood pressure before pregnancy, with elevated blood pressure (SBP ≥140 mmHg or DBP ≥90 mmHg) occurring after 20 weeks of gestation on at least two occasions separated by ≥4 h, accompanied by one of the following: proteinuria (+) or greater, or 24-h urine protein >300 mg; or in the absence of proteinuria, any of the following: platelet count < 100 × 10^9^/L, liver dysfunction, renal dysfunction, pulmonary edema, new-onset headache unexplained by other conditions, or visual disturbances (1).

Secondary outcomes included the following: GDM&PE (defined as PE patients diagnosed with GDM between 24–28 weeks of gestation), LBW (birth weight < 2,500 g), and preterm birth (delivery before 37 weeks of gestation). GDM was diagnosed using a 75-g oral glucose tolerance test (OGTT) performed at 24–28 weeks of gestation. GDM was defined when any one of the following plasma glucose thresholds was met or exceeded: fasting glucose ≥5.1 mmol/L, 1-h glucose ≥10.0 mmol/L, or 2-h glucose ≥8.5 mmol/L (19). For women with regular menstrual cycles, gestational age was calculated based on the first day of the last menstrual period. For those with irregular cycles, gestational age was estimated using early ultrasound findings.

### Statistical analysis

Baseline characteristics of the study population were described according to AIP quartiles (Q1–Q4). Normally distributed continuous variables were expressed as mean ± standard deviation (SD), while skewed continuous variables were presented as median (interquartile range, IQR). Categorical variables were reported as frequencies and percentages (%).

AIP was categorized into quartiles, with the lowest quartile (Q1) serving as the reference group. Multivariable logistic regression analysis was used to assess the association between AIP and primary and secondary outcomes, with results expressed as odds ratios (ORs) and 95% confidence intervals (CIs). In accordance with the Strengthening the Reporting of Observational Studies in Epidemiology (STROBE) statement (20), two models were constructed: Model I and Model II. Model I did not adjust for any covariates, whereas Model II adjusted for age, pre-pregnancy BMI, SBP, DBP, gestational week of testing, ALT, creatinine, FPG, aspirin use, family history of hypertension, family history of diabetes, smoking, alcohol consumption, IVF, adverse pregnancy history, parity, and education level.

To further explore whether the relationship between AIP and outcomes was influenced by categorical confounders, subgroup analyses were performed stratified by age (< 35 or ≥35 years), pre-pregnancy BMI (< 24 or ≥24 kg/m^2^), and parity (nulliparous or multiparous). Multivariable logistic regression was used for subgroup analyses, and interaction effects were evaluated using likelihood ratio tests. A *P*-value >0.05 was considered to indicate no statistically significant interaction between subgroups, while *P*-value ≤ 0.05 suggested potential population-specific effects. Additionally, a generalized additive model (GAM) with smooth curve fitting was used to examine the dose-response relationship between AIP and PE.

Statistical analyses were performed using IBM SPSS software (version 21.0; IBM Corporation, Armonk, NY) and R statistical software (version 4.4.1; R Foundation for Statistical Computing, https://www.r-project.org). All statistical tests were two-sided, and a *P*-value < 0.05 was considered statistically significant. Missing data were handled using complete-case analysis. Participants with missing information on key exposure variables, outcomes, or covariates required for multivariable analyses were excluded from the corresponding analyses, and no imputation was performed.

## Results

### Baseline characteristics

Table 1 presents the baseline clinical and demographic characteristics of participants stratified by AIP quartiles. Pregnancy outcomes across AIP quartiles are presented separately in Supplementary Table S1 to improve readability. A total of 38,822 participants meeting the inclusion and exclusion criteria were evenly distributed across the four AIP quartile groups. Participants in higher AIP quartiles tended to have higher blood pressure, BMI, ALT, FPG, and a greater proportion of IVF and adverse pregnancy history. The incidence of PE ranged from 3.85% to 8.08% across AIP quartiles, with a higher frequency of PE in the highest AIP quartile than in the lowest quartile.

**Table 1:** Baseline characteristics of participants.

Characteristic	AIP quartile				*p*-value
	Q1 (-0.74, -0.24)	Q2 (-0.24, -0.08)	Q3 (-0.08, 0.07)	Q4 (0.07, 0.58)	
Participants	9,705	9,706	9,704	9,707	
Age (years)	31.11 ± 3.76	31.24 ± 3.91	31.26 ± 4.09	31.62 ± 4.32	< 0.001
BMI (kg/m^2^)	20.55 ± 2.44	21.15 ± 2.83	21.68 ± 3.11	22.41 ± 3.44	< 0.001
SBP (mmHg)	114.13 ± 11.66	114.76 ± 12.20	115.37 ± 12.64	116.40 ± 13.07	< 0.001
DBP (mmHg)	68.60 ± 9.01	69.63 ± 9.49	70.08 ± 9.76	70.94 ± 9.93	< 0.001
AIP test week	10.16 ± 1.92	10.63 ± 2.11	10.97 ± 2.23	11.54 ± 2.38	< 0.001
AIP	−0.36 ± 0.09	−0.16 ± 0.04	−0.01 ± 0.04	0.19 ± 0.11	< 0.001
ALT (U/L)	16.24 ± 11.87	17.55 ± 15.11	18.78 ± 16.72	20.14 ± 18.80	< 0.001
Creatinine (umol/L)	43.27 ± 6.01	43.50 ± 5.99	43.69 ± 5.96	43.45 ± 6.00	< 0.001
FPG (mmol/L)	4.47 ± 0.38	4.50 ± 0.40	4.52 ± 0.45	4.56 ± 0.56	< 0.001
Aspirin (%)					
No	9,576 (98.67%)	9,561 (98.51%)	9,549 (98.40%)	9,483 (97.69%)	< 0.001
15.6-7.4,-1499ptYes	129 (1.33%)	145 (1.49%)	155 (1.60%)	224 (2.31%)	
Family history of hypertension (%)					
No	8,183 (84.33%)	8,315 (85.67%)	8,304 (85.60%)	8,359 (86.15%)	0.003
15.6-7.4,-1499ptYes	1,521 (15.67%)	1,391 (14.33%)	1,397 (14.40%)	1,344 (13.85%)	
Family history of diabetes					
No	9,119 (93.97%)	9,170 (94.48%)	9,111 (93.92%)	9,105 (93.84%)	0.224
15.6-7.4,-1499ptYes	585 (6.03%)	536 (5.52%)	590 (6.08%)	598 (6.16%)	
Tobacco (%)					
No	9,118 (98.46%)	8,866 (98.27%)	8,899 (97.81%)	8,865 (98.06%)	0.009
15.6-7.4,-1499ptYes	143 (1.54%)	156 (1.73%)	199 (2.19%)	175 (1.94%)	
Alcohol (%)					
No	8,843 (95.49%)	8,654 (95.92%)	8,738 (96.04%)	8,695 (96.18%)	0.095
15.6-7.4,-1499ptYes	418 (4.51%)	368 (4.08%)	360 (3.96%)	345 (3.82%)	
Parity (%)					
Primipara	7,903 (81.43%)	7,459 (76.85%)	7,085 (73.01%)	6,535 (67.32%)	< 0.001
15.6-7.4,-1499ptMultipara	1,802 (18.57%)	2,247 (23.15%)	2,619 (26.99%)	3,172 (32.68%)	
IVF (%)					
No	9,274 (95.56%)	9,160 (94.37%)	9,160 (94.39%)	9,002 (92.74%)	< 0.001
15.6-7.4,-1499ptYes	431 (4.44%)	546 (5.63%)	544 (5.61%)	705 (7.26%)	
Adverse pregnancy history (%)					
No	9,376 (96.61%)	9,262 (95.43%)	9,292 (95.75%)	9,179 (94.56%)	< 0.001
15.6-7.4,-1499ptYes	329 (3.39%)	444 (4.57%)	412 (4.25%)	528 (5.44%)	
Education (%)					
Postgraduate	2,680 (28.73%)	2,175 (23.90%)	1,862 (20.60%)	1,445 (16.28%)	< 0.001
Bachelor's degree or above	4,520 (48.46%)	4,357 (47.87%)	4,341 (48.03%)	3,917 (44.13%)	
College diploma	1,415 (15.17%)	1,720 (18.90%)	1,948 (21.55%)	2,393 (26.96%)	
High school	172 (1.84%)	251 (2.76%)	298 (3.30%)	413 (4.65%)	
Less than junior high school	541 (5.80%)	598 (6.57%)	589 (6.52%)	708 (7.98%)	

Percentages were calculated among participants with available data for each variable.

AIP, atherogenic index of plasma; Q, quartile; BMI, pre-pregnancy body mass index; SBP, systolic blood pressure; DBP, diastolic blood pressure; ALT, alanine aminotransferase; FPG, fasting plasma glucose; PE, preeclampsia; GDM&PE, gestational diabetes mellitus with preeclampsia; IVF, in vitro fertilization.

### Association between AIP and PE

**Figure 2:**
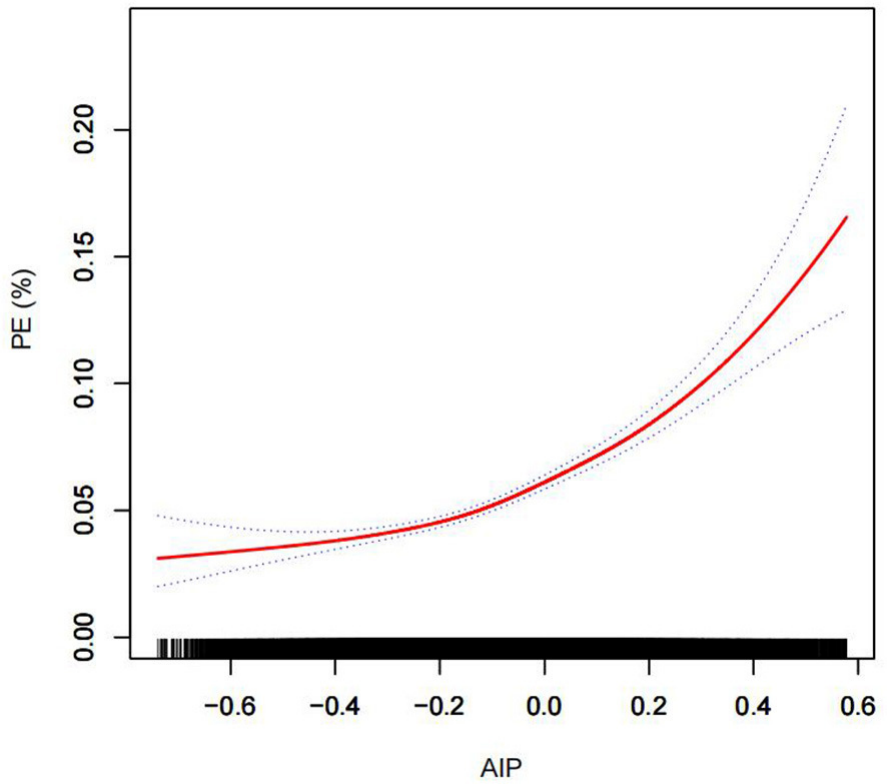
Smooth curve fitting showing the nonlinear relationship between AIP and the risk of PE. The red solid line represents the probability of PE occurrence, and the blue dotted line indicates the 95% confidence interval (CI) curve.

Table 2 summarizes the associations between AIP and the primary outcome (PE) as well as secondary outcomes (GDM&PE, preterm birth, and LBW). Stratified by AIP quartiles, with the lowest quartile serving as the reference group, multivariable regression analysis revealed that, in Model I, higher AIP values were significantly associated with an increased risk of PE, GDM&PE, and preterm birth, while a reduced risk of LBW was observed. In Model II, which accounted for potential confounders including age, BMI, SBP, and DBP, only participants in Q3 and Q4 demonstrated a significant association with increased risks of PE and GDM&PE. Compared with the first quartile of AIP, the adjusted ORs (95% CI) for PE in the third and fourth quartiles were 1.20 (1.04–1.40) and 1.43 (1.23–1.66), respectively. For GDM&PE, the corresponding adjusted ORs (95% CI) were 1.47 (1.03–2.10) and 1.81 (1.27–2.57), respectively. No significant associations were observed for preterm birth or LBW. Furthermore, when AIP was treated as a continuous variable, the Model I showed that each 0.1 increase in AIP was associated with a 17% increase in PE risk and a 31% increase in GDM&PE risk. In Model II, each 0.1 increase in AIP corresponded to an 8% increase in PE risk (OR = 1.08, 95% CI: 1.05–1.10) and a 13% increase in GDM&PE risk (OR = 1.13, 95% CI: 1.07, 1.19). The adjusted effect sizes were therefore modest. Additionally, results from smooth curve fitting indicated a nonlinear dose-response relationship between AIP and PE risk (Figure 2). These findings suggest that elevated AIP is associated with PE and GDM&PE risk, but the results should be interpreted as associations rather than evidence of standalone predictive performance.

**Table 2:** The associations between AIP and risk of primary and secondary outcomes.

Outcome	Number (%)	Model I		Model II	
		OR (95% CI)	*p*-value	Adjust OR (95% CI)	*p*-value
Primary outcome					
PE					
Q1	374 (3.85%)	Reference		Reference	
Q2	419 (4.32%)	1.13 (0.98, 1.30)	0.1032	0.94 (0.81, 1.10)	0.4604
Q3	585 (6.03%)	1.60 (1.40, 1.83)	< 0.0001	1.20 (1.04, 1.40)	0.0146
Q4	784 (8.08%)	2.19 (1.93, 2.49)	< 0.0001	1.43 (1.23, 1.66)	0.0011
Continuous AIP per 0.1		1.17 (1.14, 1.19)	< 0.0001	1.08 (1.05, 1.10)	< 0.0001
Secondary outcomes					
GDM&PE					
Q1	51 (0.53%)	Reference		Reference	
Q2	72 (0.74%)	1.41 (0.99, 2.03)	0.0588	0.98 (0.66, 1.44)	0.9055
Q3	122 (1.26%)	2.41 (1.74, 3.35)	< 0.0001	1.47 (1.03, 2.10)	0.0357
Q4	211 (2.17%)	4.21 (3.09, 5.72)	< 0.0001	1.81 (1.27, 2.57)	0.0009
Continuous AIP per 0.1		1.31 (1.26, 1.37)	< 0.0001	1.13 (1.07, 1.19)	< 0.0001
Preterm birth					
Q1	432 (4.45%)	Reference		Reference	
Q2	495 (5.10%)	1.15 (1.01, 1.32)	0.0341	1.10 (0.96, 1.27)	0.1654
Q3	528 (5.44%)	1.24 (1.08, 1.41)	0.0015	1.13 (0.98, 1.30)	0.0852
Q4	573 (5.90%)	1.35 (1.18, 1.53)	< 0.0001	1.12 (0.97, 1.29)	0.1353
Continuous AIP per 0.1		1.06 (1.03, 1.08)	< 0.0001	1.02 (1.00, 1.04)	0.1003
Low birth weight					
Q1	447 (4.61%)	Reference		Reference	
Q2	426 (4.39%)	0.95 (0.83, 1.09)	0.4642	0.95 (0.83, 1.10)	0.5024
Q3	433 (4.46%)	0.97 (0.84, 1.11)	0.6258	1.02 (0.88, 1.18)	0.8162
Q4	371 (3.82%)	0.82 (0.71, 0.95)	0.0066	0.87 (0.74, 1.01)	0.0735
Continuous AIP per 0.1		0.97 (0.95, 0.99)	0.0070	0.98 (0.95, 1.00)	0.1098

Model I: No covariates were adjusted.

Model II: Adjusted for age, BMI, SBP, DBP, test week, ALT, creatinine, FPG, aspirin, family history of hypertension, family history of diabetes, tobacco, alcohol, IVF, adverse pregnancy history, parity, and education levels.

PE, preeclampsia; GDM&PE, gestational diabetes mellitus with preeclampsia; AIP, atherogenic index of plasma; Q, quartile; OR, odds ratio; CI, confidence interval.

### Sensitivity analysis

To further confirm the relationship between AIP and PE as well as GDM&PE, subgroup analyses were conducted. As shown in Table 3, after adjusting for confounders, stratification by pre-pregnancy BMI revealed consistent associations between AIP quartiles and PE. However, among pregnant women aged < 35 years and nulliparous women, the association between AIP and PE was more pronounced in Q3 and Q4 compared to Q1. Similarly, the association between AIP quartiles and GDM&PE remained consistent across subgroups defined by age < 35 years, BMI ≥ 24, and nulliparity, demonstrating the robustness of the findings (Table 4). When AIP was treated as a continuous variable, similar trends were observed. Interaction analyses indicated robust results across different subgroups for the associations between AIP and PE or GDM&PE (*P* for interaction > 0.05).

**Table 3:** Subgroup analysis of the AIP index and preeclampsia.

Subgroup	AIP quartile	Non-adjusted OR (95% CI)	*p*-value	*p*-value for interaction	Adjusted OR (95% CI)	*p*-value	*p*-value for interaction
Age < 35	Q1	Reference		0.4270	Reference		0.4557
	Q2	1.13 (0.97, 1.33)	0.1263		0.96 (0.81, 1.14)	0.6721	
	Q3	1.54 (1.33, 1.79)	< 0.0001		1.18 (0.99, 1.39)	0.0576	
	Q4	2.20 (1.91, 2.54)	< 0.0001		1.47 (1.24, 1.73)	< 0.0001	
Age≥35	Q1	Reference			Reference		
	Q2	1.06 (0.77, 1.46)	0.7211		0.85 (0.58, 1.23)	0.3801	
	Q3	1.70 (1.27, 2.27)	0.0004		1.29 (0.92, 1.82)	0.1423	
	Q4	1.95 (1.48, 2.59)	< 0.0001		1.28 (0.91, 1.79)	0.1558	
Age < 35	Continuous AIP per 0.1	1.16 (1.14, 1.19)	< 0.0001	0.825	1.08 (1.05, 1.11)	< 0.0001	0.9122
Age≥35	Continuous AIP per 0.1	1.16 (1.11, 1.21)	< 0.0001		1.07 (1.02, 1.13)	0.0073	
BMI < 24	Q1	Reference		0.4181	Reference		0.5514
	Q2	1.02 (0.87, 1.20)	0.8130		0.92 (0.77, 1.10)	0.3576	
	Q3	1.28 (1.09, 1.50)	0.0021		1.11 (0.93, 1.31)	0.2542	
	Q4	1.69 (1.45, 1.97)	< 0.0001		1.39 (1.17, 1.65)	0.0002	
BMI≥24	Q1	Reference			Reference		
	Q2	1.14 (0.83, 1.55)	0.4268		0.98 (0.69, 1.39)	0.8902	
	Q3	1.64 (1.23, 2.18)	0.0007		1.42 (1.03, 1.96)	0.0335	
Q4	1.87 (1.42, 2.46)	< 0.0001		1.52 (1.11, 2.08)	0.0097	
BMI < 24	Continuous AIP per 0.1	1.11 (1.08, 1.14)	< 0.0001	0.3805	1.06 (1.03, 1.09)	< 0.0001	0.198
BMI≥24	Continuous AIP per 0.1	1.13 (1.09, 1.17)	< 0.0001		1.10 (1.05, 1.15)	< 0.0001	
Primipara	Q1	Reference		0.7466	Reference		0.1591
	Q2	1.17 (1.01, 1.37)	0.0393		0.98 (0.83, 1.16)	0.8582	
	Q3	1.65 (1.43, 1.91)	< 0.0001		1.18 (1.01, 1.39)	0.0415	
	Q4	2.37 (2.07, 2.73)	< 0.0001		1.48 (1.26, 1.74)	< 0.0001	
Multipara	Q1	Reference			Reference		
	Q2	1.02 (0.68, 1.51)	0.9365		0.64 (0.41, 1.00)	0.0497	
	Q3	1.73 (1.22, 2.46)	0.0023		1.18 (0.79, 1.75)	0.4144	
	Q4	2.25 (1.61, 3.14)	< 0.0001		1.07 (0.72, 1.58)	0.7465	
Primipara	Continuous AIP per 0.1	1.18 (1.16, 1.21)	< 0.0001	0.8607	1.08 (1.05, 1.11)	< 0.0001	0.3984
Multipara	Continuous AIP per 0.1	1.19 (1.13, 1.25)	< 0.0001		1.05 (0.99, 1.11)	0.0992	

Non-adjusted model had no adjustments; Each stratification was adjusted for age, BMI, SBP, DBP, test week, ALT, creatinine, FPG, aspirin, family history of hypertension, family history of diabetes, tobacco, alcohol, IVF, adverse pregnancy history, parity, and education levels, except the stratification factor itself. PE, preeclampsia; AIP, atherogenic index of plasma; Q, quartile; BMI, pre-pregnancy body mass index; OR, odds ratio; CI, confidence interval.

**Table 4:** Subgroup analysis of the AIP index and GDM&PE.

Subgroup	AIP quartile	Non-adjusted OR (95% CI)	*p*-value	*p*-value for interaction	Adjusted OR (95% CI)	*p*-value	*p*-value for interaction
Age < 35	Q1	Reference		0.1614	Reference		0.0495
	Q2	1.59 (1.03, 2.43)	0.0342		1.20 (0.76, 1.88)	0.4330	
	Q3	2.31 (1.55, 3.46)	< 0.0001		1.41 (0.92, 2.18)	0.1185	
	Q4	4.59 (3.17, 6.65)	< 0.0001		2.03 (1.34, 3.08)	0.0009	
Age≥35	Q1	Reference			Reference		
	Q2	0.98 (0.50, 1.93)	0.9576		0.51 (0.23, 1.15)	0.1041	
	Q3	2.26 (1.27, 4.01)	0.0055		1.47 (0.77, 2.81)	0.2476	
	Q4	2.80 (1.61, 4.86)	0.0002		1.30 (0.68, 2.48)	0.4309	
Age < 35	Continuous AIP per 0.1	1.33 (1.26, 1.40)	< 0.0001	0.0995	1.15 (1.08, 1.22)	< 0.0001	0.4001
Age≥35	Continuous AIP per 0.1	1.23 (1.14, 1.33)	< 0.0001		1.09 (0.99, 1.20)	0.0637	
BMI < 24	Q1	Reference		0.0238	Reference		0.064
	Q2	1.12 (0.73, 1.71)	0.6145		0.89 (0.56, 1.42)	0.6329	
	Q3	1.19 (0.78, 1.83)	0.4159		0.96 (0.60, 1.53)	0.8656	
	Q4	2.07 (1.40, 3.05)	0.0003		1.48 (0.96, 2.30)	0.0781	
BMI≥24	Q1	Reference			Reference		
	Q2	1.75 (0.85, 3.61)	0.1303		1.35 (0.61, 2.96)	0.4589	
	Q3	3.46 (1.78, 6.71)	0.0002		2.76 (1.34, 5.65)	0.0056	
	Q4	4.55 (2.38, 8.68)	< 0.0001		2.83 (1.39, 5.75)	0.0040	
BMI < 24	Continuous AIP per 0.1	1.15 (1.07, 1.23)	< 0.0001	0.0554	1.07 (1.00, 1.16)	0.0599	0.1196
BMI≥24	Continuous AIP per 0.1	1.25 (1.18, 1.33)	< 0.0001		1.17 (1.08, 1.26)	< 0.0001	
Primipara	Q1	Reference		0.7734	Reference		0.852
	Q2	1.43 (0.98, 2.10)	0.0661		1.01 (0.67, 1.53)	0.9562	
	Q3	2.47 (1.74, 3.51)	< 0.0001		1.43 (0.97, 2.10)	0.0678	
	Q4	4.23 (3.04, 5.89)	< 0.0001		1.78 (1.22, 2.60)	0.0025	
Multipara	Q1	Reference			Reference		
	Q2	1.61 (0.55, 4.71)	0.3875		0.72 (0.22, 2.40)	0.5957	
	Q3	2.91 (1.09, 7.72)	0.0324		1.74 (0.62, 4.89)	0.2967	
	Q4	6.11 (2.44, 15.31)	0.0001		1.92 (0.70, 5.26)	0.2025	
Primipara	Continuous AIP per 0.1	1.33 (1.27, 1.40)	< 0.0001	0.7178	1.13 (1.07, 1.20)	< 0.0001	0.7444
Multipara	Continuous AIP per 0.1	1.36 (1.23, 1.50)	< 0.0001		1.11 (0.99, 1.25)	0.0842	

Non-adjusted model had no adjustments; Each stratification was adjusted for age, BMI, SBP, DBP, test week, ALT, creatinine, FPG, aspirin, family history of hypertension, family history of diabetes, tobacco, alcohol, IVF, adverse pregnancy history, parity, and education levels, except the stratification factor itself. GDM&PE, gestational diabetes mellitus with preeclampsia; AIP, atherogenic index of plasma; Q, quartile; BMI, pre-pregnancy body mass index; OR, odds ratio; CI, confidence interval.

**Figure 3:**
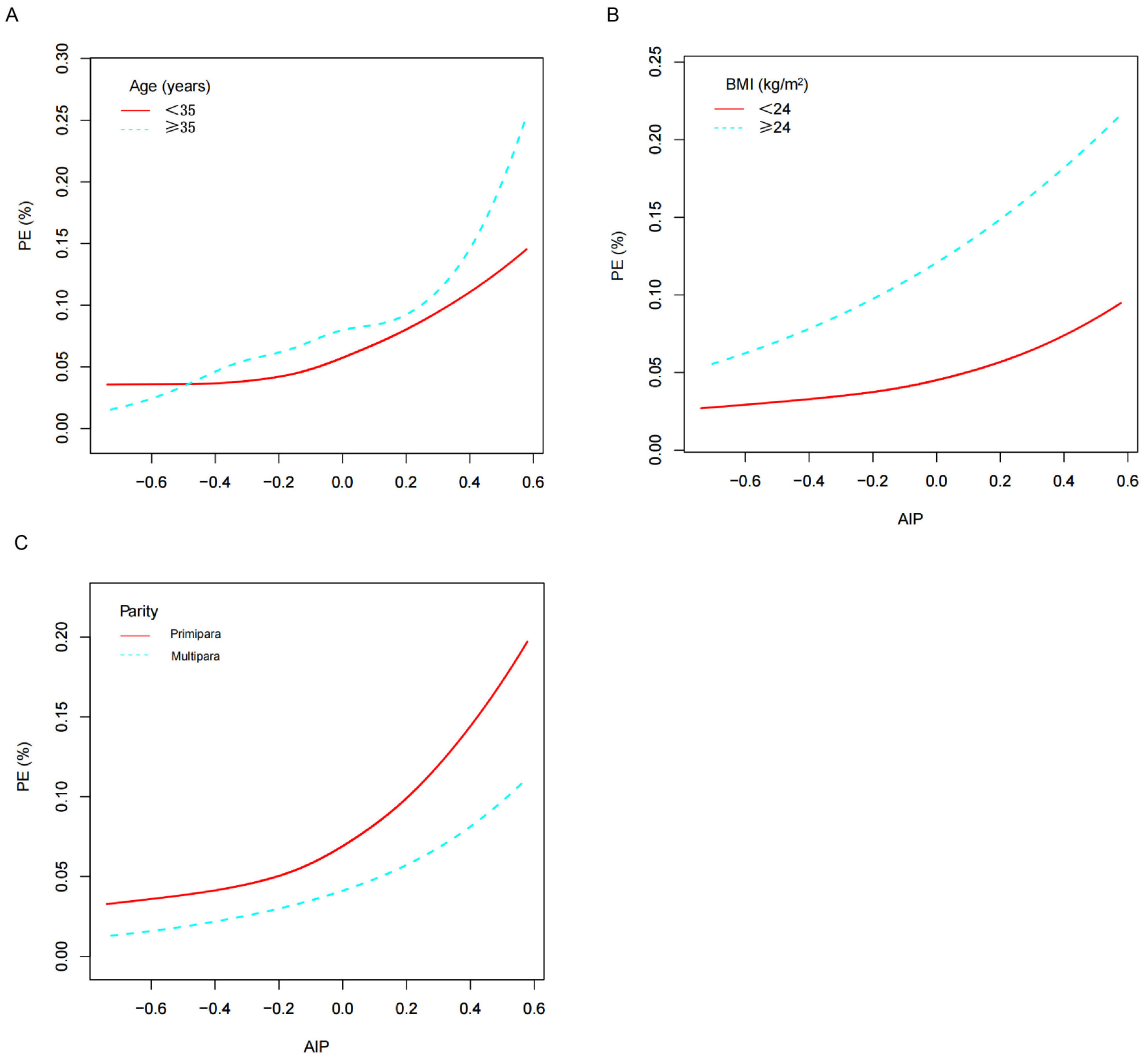
The dose-response relationship between AIP and the risk of PE, stratified by age **(A)**, BMI **(B)**, and parity **(C)**.

Figure 3 illustrates the dose-response relationship between AIP and PE risk derived from smooth curve fitting. The results indicate that the incidence of PE increased significantly with higher AIP levels. Specifically, across different age groups (< 35 years and ≥ 35 years), BMI categories (< 24 and ≥ 24), and parity groups (nulliparous and multiparous), the risk of PE rose with increasing AIP levels. This finding further supports the association between elevated AIP and increased PE risk.

## Discussion

PE is a pregnancy-specific complication that poses a significant threat to maternal and neonatal health. Dyslipidemia has been associated with the development of PE, and AIP, as a novel lipid metabolism marker, comprehensively reflects the balance between pro-atherogenic and anti-atherogenic lipoproteins. However, its relationship with PE remains unclear. Based on multicenter clinical and laboratory data, this study systematically investigated the associations between AIP and PE, as well as related adverse pregnancy outcomes. The findings revealed a positive correlation between AIP and the risks of PE and GDM&PE. Even after adjusting for potential confounders, higher AIP levels were significantly associated with increased risks of PE and GDM&PE, although the adjusted effect sizes were modest. When AIP was treated as a continuous variable, each 0.1 increase in AIP corresponded to an 8% increase in PE risk and a 13% increase in GDM&PE risk after adjustment for confounders. Subgroup analyses further showed that the association between AIP and PE was not affected by maternal age, BMI, or parity. Additionally, a nonlinear dose-response relationship was observed between AIP and PE risk. These findings suggest that AIP may serve as a potential risk marker for PE, but they do not establish AIP as a standalone screening or diagnostic biomarker.

During pregnancy, maternal lipid levels gradually increase, particularly TG and cholesterol, providing essential nutritional support for fetal growth and development. This lipid alteration is considered physiological (21). However, when dyslipidemia (e.g., elevated TG and reduced HDL) exceeds a certain threshold, it may lead to atherosclerosis and is associated with various pregnancy complications, including PE and GDM (22, 23). Studies have shown that compared to normal pregnancies, PE patients exhibit significantly elevated TC, TG, and low-density lipoprotein cholesterol (LDL-C), along with markedly reduced HDL (8). A prospective cohort study also found that PE patients exhibited elevated TC, TG, and LDL-C levels even in early pregnancy (24). In contrast, Hentschke et al. did not observe significant differences in lipid profiles between PE and normal pregnancies (25). The present study demonstrated that elevated AIP before 20 weeks of gestation was associated with an increased risk of PE, suggesting that dyslipidemia may be involved in PE risk. This finding aligns with most previous studies. Unlike prior studies that focused on single lipid parameters, this study utilized the composite lipid index AIP to evaluate maternal-fetal lipid abnormalities. By incorporating both TG and HDL-C, AIP not only reflects their ratio but also captures interactions between different lipid components, thereby providing a more integrated representation of dyslipidemia (26). Notably, compared to AIP Q1, AIP Q2 was not significantly associated with PE, whereas AIP Q3 (AIP = −0.08 to 0.07) and AIP Q4 (AIP = 0.07 to 0.58) showed significant associations. This suggests that mild lipid changes may represent a physiological adaptation to meet fetal growth demands, while more pronounced TG elevation and HDL-C reduction may reflect pathological metabolic disturbance related to PE. Given the lack of a unified threshold for pathological dyslipidemia in pregnancy, our findings may provide a reference for future studies. Early identification of women with pronounced dyslipidemia may support closer monitoring and risk-factor counseling, but interventional studies are needed before inferring that modifying AIP can prevent PE. Future research should compare the predictive performance and incremental value of AIP with other lipid markers and clinical risk factors in adverse pregnancy outcomes.

Subgroup analyses showed that the positive association between AIP and PE was generally observed across pre-pregnancy BMI subgroups. When stratified by maternal age and parity, the association appeared more evident among women aged < 35 years and nulliparous women. This finding is partly consistent with previous studies suggesting that younger maternal age may be associated with elevated liver enzymes and dyslipidemia during pregnancy (27). One possible explanation is that younger and nulliparous women may have a lower prevalence of established PE risk factors, such as chronic hypertension and diabetes; therefore, early-pregnancy lipid abnormalities may be more readily observed in relation to PE risk in these subgroups. However, the interaction tests for maternal age and parity were not statistically significant. Therefore, these stratified findings should be interpreted cautiously and should be considered exploratory rather than definitive evidence of effect modification by maternal age or parity. Further studies with prespecified subgroup hypotheses and adequate statistical power are needed to validate these observations.

Regarding secondary outcomes, we found that elevated AIP before 20 weeks of gestation was associated with an increased risk of GDM&PE but not with preterm birth or LBW. Previous studies have shown that dyslipidemia is closely linked to GDM. Elevated TG levels in early pregnancy significantly increase the risk of GDM; in particular, women with TG concentrations ≥137 mg/dl had a 3.5-fold higher risk of GDM. In addition, reduced HDL-C levels have also been associated with an increased risk of GDM (28, 29). Multiple studies have further confirmed the association between AIP and diabetes. For example, a cross-sectional study of 10,099 American adults found that each unit increase in AIP was associated with a 4.96-fold higher prevalence of prediabetes and diabetes in women (30). Another study involving 100,069 Chinese adults showed a positive correlation between AIP and the incidence of prediabetes (31). Recent prospective studies have also identified AIP as an independent risk factor for GDM (32). Nevertheless, the performance of early-pregnancy metabolic indices as standalone predictive tools appears limited. A recent prospective diagnostic-accuracy study evaluating first-trimester inflammatory markers, HDL-based ratios, AIP, and TyG reported that AIP and TyG alone had limited value for predicting GDM and should not replace OGTT or broader risk assessment (33). This evidence supports a balanced interpretation of the present findings: AIP may contribute to risk stratification when combined with clinical characteristics and other metabolic indicators, but it should not be regarded as a clinically validated standalone predictive biomarker. Unlike previous studies, this research is the first to reveal the association between AIP and GDM&PE. GDM and PE, as metabolic-related complications, share common pathophysiological mechanisms, including insulin resistance, endothelial dysfunction, inflammatory responses, and placental dysfunction (34–37). Dyslipidemia may increase the risks of PE and GDM through these mechanisms, while the presence of GDM may further exacerbate dyslipidemia, thereby promoting PE development. Despite the established associations among dyslipidemia, PE, and GDM, the precise mechanisms remain to be elucidated. Furthermore, no significant association was observed between AIP and LBW in the present study. Previous findings on the relationship between maternal lipid metabolism abnormalities and newborn birth weight have been inconsistent. Some studies have reported that elevated TG or reduced HDL-C in late pregnancy is associated with an increased risk of LBW (38), while others suggest that only low HDL is associated with LBW, with no significant effects from other lipid parameters (39). Another study indicated that dyslipidemia in early pregnancy is not related to LBW, whereas dyslipidemia in late pregnancy is positively correlated with newborn birth weight (40). These discrepancies suggest that the impact of maternal lipids on fetal weight is complex. Theoretically, maternal TG can be hydrolyzed into free fatty acids (FFAs) in the placenta via lipoprotein lipase, and FFAs entering the fetal circulation may lead to fat and glucose deposition, causing fetal hyperlipidemia and hyperglycemia, which may ultimately result in increased fetal weight. Additionally, maternal cholesterol can be transported across the placenta and regulate fetal cholesterol synthesis, influencing fetal size and weight. However, dyslipidemia may impair placental function, leading to insufficient nutrient supply to the fetus. The specific roles of these mechanisms require further investigation.

Several explanations have been proposed to elucidate the mechanisms by which dyslipidemia influences the development of PE. Endothelial dysfunction is a shared mechanism in both atherosclerosis and the pathogenesis of PE, with dyslipidemia directly contributing to endothelial dysfunction. During pregnancy, excessive TG accumulate in the endothelial cells of uterine spiral arteries, reducing prostacyclin synthesis while increasing endothelin synthesis, thereby promoting vasoconstriction and leading to endothelial dysfunction (41). Additionally, TG is hydrolyzed into FFAs, which bind to apolipoprotein-CIII, enhancing oxidative stress in endothelial cells and driving the progression of atherosclerosis (42). Apolipoprotein A-1, the major protein component of HDL, exerts an opposing effect to TG by promoting cholesterol efflux from arterial wall cells, thereby regulating vascular endothelial function (43). Studies have shown that reduced HDL levels and functional alterations in PE patients impair paraoxonase 1 activity, contributing to endothelial dysfunction (44). Another potential mechanism involves the overactivation of inflammatory immune responses. While moderate inflammation is essential for maintaining maternal-fetal immune tolerance, excessive inflammatory activation can trigger endothelial dysfunction and maternal vascular damage, thereby promoting PE development (45). Atherosclerosis, as a chronic inflammatory disease, is characterized early on by the overexpression of adhesion molecules on endothelial cells, facilitating the migration of inflammatory cells into the intima. Conversely, HDL inhibits the expression of these adhesion molecules (46). Epigenetic changes in the placenta represent another potential mechanism through which dyslipidemia contributes to PE. High TG levels are associated with abnormal DNA methylation of ALX4, a key gene involved in placental vascular remodeling (47). Low HDL levels correlate with increased placental DNA methylation at loci relevant to lipoprotein lipase and ABCA1, disrupting cholesterol transfer from mother to fetus (48). Furthermore, trophoblast dysfunction, oxidative stress, and ferroptosis also serve as mechanisms by which lipids mediate the occurrence of PE (49).

The primary strengths of this study lie in the utilization of multicenter, large-sample clinical and laboratory data, which enhances the generalizability of the findings within the study population. Through multivariable logistic regression analysis, subgroup analyses and smooth curve fitting, we comprehensively explored the relationships between AIP and PE as well as GDM&PE, while accounting for the influence of potential confounders, thereby improving the robustness of the association analyses. Additionally, AIP is derived from routine laboratory measurements and is easily accessible in clinical practice. These features support its potential value as a risk marker for early risk assessment, while further predictive-performance and calibration analyses are needed before clinical implementation.

However, several limitations should be acknowledged. First, as this was a retrospective observational study, causal relationships between AIP and PE cannot be established, and residual confounding or selection bias cannot be completely excluded. Missing data were handled using complete-case analysis, which may also have introduced potential selection bias. Second, although elevated AIP was significantly associated with PE risk, the adjusted effect sizes were modest, and predictive performance metrics, such as ROC curves, AUC, sensitivity, specificity, calibration, optimal cut-off values, and incremental value beyond established clinical risk factors, were not evaluated. Therefore, AIP should be interpreted as a potential risk marker rather than a standalone screening or diagnostic biomarker. Third, aspirin use may have introduced confounding by indication, as aspirin is commonly prescribed to women at high risk of PE. Although aspirin use was adjusted for in the multivariable model, detailed indications and exact initiation timing were not uniformly available, and a reliable sensitivity analysis excluding aspirin users could not be performed. Fourth, this study evaluated only early-pregnancy AIP and did not assess serial lipid changes during pregnancy. In addition, PE was analyzed as an overall outcome without stratification by clinical phenotype, such as early- versus late-onset PE, which may have introduced biological heterogeneity. Finally, unmeasured factors, such as diet and physical activity, may still have influenced the results. Future studies incorporating serial lipid trajectories, PE phenotype-specific analyses, lifestyle factors, and predictive performance assessments are warranted.

## Conclusions

Elevated AIP in early pregnancy was associated with increased risks of PE and GDM&PE in this retrospective observational cohort. AIP may serve as a potential risk marker for early PE risk stratification, but the observed associations were modest and do not establish AIP as a standalone screening or diagnostic biomarker. Further prospective studies with serial measurements and predictive-performance analyses are needed to determine its incremental clinical value.

## Data Availability

The raw data supporting the conclusions of this article will be made available by the authors, without undue reservation.
