# Correction: Association between atherogenic index of plasma in early pregnancy and risk of preeclampsia: a multicenter cohort study

**DOI:** 10.3389/fnut.2026.1927178

**Published:** 2026-07-22

**Authors:** Qiong Li, Yuping Duan, Chenyang Zhao, Ying Gu, Yanfei Qian, Chaoyan Yue

**Affiliations:** 1 Department of Obstetrics and Gynecology, The First People's Hospital of Chenzhou, Hengyang Medical School, University of South China, Chenzhou, China; 2 Department of Clinical Laboratory, Wusong Central Hospital, Baoshan, Shanghai, China; 3 Department of Obstetrics, Women's Hospital of Jiangnan University, Wuxi Maternity and Child Health Care Hospital, Wuxi, China; 4 Department of Clinical Laboratory, Children's Hospital of Fudan University, Shanghai, China; 5 Obstetrics & Gynecology Hospital of Fudan University, Shanghai Key Lab of Reproduction and Development, Shanghai Key Lab of Female Reproductive Endocrine Related Diseases, Shanghai, China

**Keywords:** atherogenic index of plasma, lipids, multicenter cohort study, preeclampsia, risk marker

In the **Funding** statement, the funders “Baoshan District Health Commission Excellent Youth (Yucai) Program (BSWSYC-20232208)” were omitted.

The corrected **Funding** statement is as follows:

“The author(s) declared that financial support was received for this work and/or its publication. This study was supported by the Natural Science Foundation of Hunan Province (2026JJ80605), Baoshan District Health Commission Excellent Youth (Yucai) Program (BSWSYC-20232208), Shanghai Baoshan District Medical and Health Project Fund (2025-E-32), and the Natural Science Foundation of Shanghai Basic Research Program (25ZR1402039).”

The original version of this article has been updated.

