# Supplementary material for: Association between atherogenic index of plasma in early pregnancy and risk of preeclampsia: a multicenter cohort study
Source: Front Nutr. 2026 Jun 18;13:1849933. doi: 10.3389/fnut.2026.1849933 (PMC13323682; doi:10.3389/fnut.2026.1849933)
Supplement: Supplementary file 1 [file Table_1.doc]

Supplementary Table S1 Distribution of pregnancy outcomes across AIP quartiles.

| Outcome | AIP quartile |  |  |  |  |
| --- | --- | --- | --- | --- | --- |
|  | Q1 (-0.74, -0.24) | Q2 (-0.24,-0.08) | Q3 (-0.08, 0.07) | Q4 (0.07, 0.58) | P-value |
| PE (%) |  |  |  |  | <0.001 |
| No | 9331 (96.15%) | 9287 (95.68%) | 9119 (93.97%) | 8923 (91.92%) |  |
| Yes | 374 (3.85%) | 419 (4.32%) | 585 (6.03%) | 784 (8.08%) |  |
| GDM&PE |  |  |  |  | <0.001 |
| No | 9654 (99.47%) | 9634 (99.26%) | 9582 (98.74%) | 9496 (97.83%) |  |
| Yes | 51 (0.53%) | 72 (0.74%) | 122 (1.26%) | 211 (2.17%) |  |
| Preterm birth (%) |  |  |  |  | <0.001 |
| No | 9273 (95.55%) | 9210 (94.90%) | 9176 (94.56%) | 9134 (94.10%) |  |
| Yes | 432 (4.45%) | 495 (5.10%) | 528 (5.44%) | 573 (5.90%) |  |
| Low birth weight (%) |  |  |  |  | 0.039 |
| No | 9253 (95.39%) | 9277 (95.61%) | 9270 (95.54%) | 9334 (96.18%) |  |
| Yes | 447 (4.61%) | 426 (4.39%) | 433 (4.46%) | 371 (3.82%) |  |
